# Quantitative imaging analysis detects subtle airway abnormalities in symptomatic military deployers

**DOI:** 10.1186/s12890-022-01960-w

**Published:** 2022-04-27

**Authors:** Lauren M. Zell-Baran, Stephen M. Humphries, Camille M. Moore, David A. Lynch, Jean-Paul Charbonnier, Andrea S. Oh, Cecile S. Rose

**Affiliations:** 1 Division of Environmental and Occupational Health Sciences, National Jewish Health, Denver, CO USA; 2 Department of Radiology, National Jewish Health, Denver, CO USA; 3 School of Medicine, University of Colorado, Aurora, CO USA; 4 Center for Genes, Environment and Health, National Jewish Health, Denver, CO USA; 5 Department of Biostatistics and Informatics, University of Colorado, Aurora, CO USA; 6 Thirona, Nijmegen, Netherlands

**Keywords:** Military deployment, HRCT, Quantitative image analysis, Emphysema, Bronchial wall thickening, Air trapping

## Abstract

**Background:**

Exposure to inhalational hazards during post-9/11 deployment to Southwest Asia and Afghanistan puts military personnel at risk for respiratory symptoms and disease. Pulmonary function and qualitative chest high resolution computed tomography (HRCT) are often normal in “deployers” with persistent respiratory symptoms. We explored the utility of quantitative HRCT imaging markers of large and small airways abnormalities, including airway wall thickness, emphysema, and air trapping, in symptomatic deployers with clinically-confirmed lung disease compared to controls.

**Methods:**

Chest HRCT images from 45 healthy controls and 82 symptomatic deployers with asthma, distal lung disease or both were analyzed using Thirona Lung quantification software to calculate airway wall thickness (by Pi10), emphysema (by percentage of lung volume with attenuation < -950 Hounsfield units [LAA%-950]), and three parameters of air trapping (expiratory/inspiratory total lung volume and mean lung density ratios, and LAA%-856). SAS v.9.4 was used to compare demographic and clinical characteristics between deployers and controls using Chi-Square, Fisher Exact or t-tests. Linear regression was used to assess relationships between pulmonary function and quantitative imaging findings.

**Results:**

Gender and smoking status were not statistically significantly different between groups, but deployers were significantly younger than controls (42 vs 58 years, p < 0.0001), had higher body mass index (31 vs 28 kg/m^2^, p = 0.01), and had fewer total smoking pack-years (8 vs. 26, p = 0.007). Spirometric measures were not statistically significantly different between groups. Pi10 and LAA%-950 were significantly elevated in deployers compared to controls in unadjusted analyses, with the emphysema measure remaining significantly higher in deployers after adjustment for age, sex, smoking, BMI, and expiratory total lung volume. Air trapping parameters were more common in control images, likely due to differences in age and smoking between groups. Among deployers, LAA%-950 and Pi10 were significantly correlated with spirometric markers of obstruction based on ratio of forced expiratory volume in one second (FEV1)/forced vital capacity (FVC) and/or percent predicted FEV1.

**Conclusions:**

Quantitative chest HRCT imaging analysis identifies emphysema in deployers with asthma and distal lung disease, and may be useful in detecting and monitoring deployment-related lung disease in a population where spirometry is typically normal.

## Background

Inhalation of hazardous particulate matter from burn pit emissions, desert dust, occupational vapors, dusts, gases, and fumes (VDGF), explosive blasts, and diesel exhaust during deployment to Iraq, Afghanistan, and other Southwest Asia locations may place deployed military personnel (‘deployers’) at increased risk for respiratory diseases [1–5]. Deployment-related asthma, bronchiolitis, and persistent, sometimes career-ending, respiratory symptoms have been reported in those who deployed to these hazardous environments since September 11, 2001 [6–14]. Pulmonary function tests and qualitative visual assessment of high resolution computed tomography (HRCT) findings are often normal or nonspecific, and more sensitive diagnostic markers of lung disease are needed.

Quantitative analyses of HRCT, including measures of lung density to evaluate emphysema and airway metrics to quantify bronchial wall thickening, are sensitive markers of disease in those with chronic obstructive pulmonary disease (COPD), asthma, interstitial lung disease, and cardiovascular disease [15–25]. These measures were significantly more sensitive than spirometry in classifying disease phenotypes (airways predominant, emphysema predominant, mixed) among patients with COPD [15]. Moreover, airway wall thickness and emphysema measurements on HRCT have been associated with bronchodilator responsiveness, St. George's Respiratory Questionnaire (SGRQ) scores, BODE (Body-mass, airflow Obstruction, Dyspnea, and Exercise) Index scores, pulmonary function testing measurements, dyspnea score/severity, and pulmonary arterial pressure [16–26].

In a large COPD cohort study (SPIROMICS), analysis of occupational exposure data in 2736 participants (1809 with COPD and 927 without airflow limitation) showed that those with VDGF exposure in their longest held job (49%) had significantly greater airway wall thickness, expressed as Pi10, as well as higher odds of emphysema and large and small airways disease [27]. We hypothesized that symptomatic military personnel with exposure to complex inhalational hazards during deployment would have abnormal measures of airway wall thickness, emphysema, and air trapping on quantitative imaging analysis compared to controls. To evaluate the utility of quantitative imaging analysis in diagnosis of deployment-related lung disease, we compared findings in those with and without clinically confirmed asthma and/or histologically verified distal lung disease. Additionally, we explored whether pulmonary function parameters associated with airways diseases would be associated with more abnormal quantitative imaging findings.

## Methods

### Study populations

**Fig. 1:**
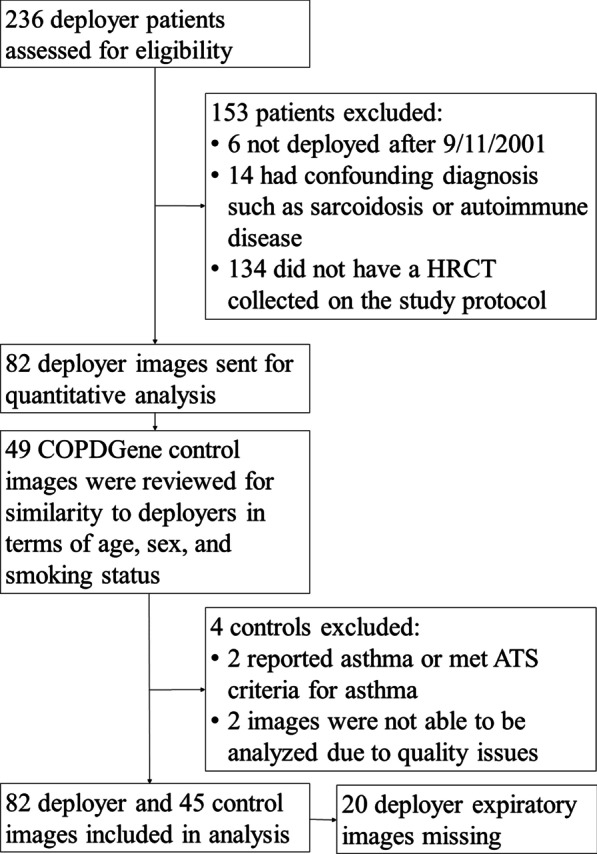
Flow diagram for inclusion in this observational cross-sectional study

We conducted a cross sectional study using 82 deployer and 45 control chest HRCT images (see Fig. 1). With ethics committee approval and informed consent (HS-2689/HS-3022), we obtained images from patients seen in the Center for Deployment-Related Lung Disease at National Jewish Health who were evaluated for persistent respiratory symptoms that began during or after post-9/11 deployment to Southwest Asia. Using a standardized questionnaire, we collected information on medical and smoking histories. Clinical testing included pre- and post-bronchodilator body plethysmographic pulmonary function testing (including residual lung volumes [RV], spirometry, total lung capacity [TLC], and diffusion capacity for carbon monoxide [DLCO]), methacholine challenge, and chest HRCT imaging.

Pulmonary function and methacholine challenge testing were conducted in accordance with American Thoracic Society (ATS) Standards [28–31] and analyzed along with the most closely temporally linked HRCT. Reference values for spirometry were obtained from National Health and Nutrition Examination Survey III [32]. Lung volume reference values were based on Cherniack 1977 [33]. DLCO reference values were obtained from the Global Lung Function Initiative (GLI) prediction equations [34].

Deployers were classified as those with definite deployment-related lung disease including those with deployment-related asthma (DRA) and/or biopsy-proven deployment-related distal lung disease (DDLD, n = 24). Case definitions are detailed in a previous study [6]. Briefly, we defined biopsy-proven DDLD based on a pulmonary pathologist’s identification of abnormal distal lung histologic findings of bronchiolitis, granulomatous pneumonitis, and/or hyperinflation with emphysema on surgical lung biopsy. DRA diagnosis required either a post-bronchodilator increase in the percent predicted forced expiratory volume in the first second (FEV1_PP_) ≥ 12% and ≥ 200 ml on pulmonary function testing or airways hyper-responsiveness based on methacholine challenge [35]. Those with persistent respiratory symptoms and clinical findings who did not have DRA and who did not undergo lung biopsy were analyzed separately and are referred to as those with possible disease (i.e. possible DDLD).

Control HRCT images from smoking and non-smoking subjects with normal spirometry were obtained with permission from the COPDGene® Study (ancillary study number ANC246). Controls were selected to be as similar as possible to deployers in terms of age, gender, race/ethnicity, smoking status (ever, current, former), and smoking pack-years. As described previously [36, 37], the non-smoking controls with no history of lung disease were recruited at COPDGene centers around the United States using word-of-mouth communication to friends and spouses of COPD subjects, advertisements, and outreach to community groups and churches. Participants were between the ages of 45–80. Potential control participants who met the ATS spirometry definition for asthma (both a 200 cc and a 12% increase in FEV1_PP_ after bronchodilator) or self-reported diagnoses of asthma or other lung disease in the last five years were excluded from the current study [38].

### Imaging acquisition, qualitative assessment, and quantitative analysis

All HRCT scans were acquired using the same reduced-dose protocol as that used in Phase 3 of the COPDGene^©^ study [39]. Thin sections (0.5–0.75 mm slice thickness) with a high spatial frequency reconstruction algorithm were used to enhance parenchymal and small airways findings. Volumetric scans were obtained on full inspiration with dose modulation (modulated ref 35 mAs) and at the end of normal expiration (Functional Residual Capacity) (50 mA). A radiology analyst uploaded images in TeraRecon for independent scoring by study radiologists, who were blinded to deployer versus control scan status.

Three thoracic radiologists blinded to subject status independently scored all HRCT images. One study radiologist left National Jewish Health before completion of the study and was replaced by a fourth radiologist. Radiologists used a REDCap-based scoring form that was pilot-tested and modified to focus mainly on large and small airways findings. Variables of interest for this analysis included the presence and extent of airways abnormalities including bronchial wall thickening (absent/mild/moderate/severe), air trapping (absent/mild/moderate/severe), and emphysema (absent/present). The mode was used to calculate tie-break or consensus scores between readers. In cases where the mode did not exist, discordant readings of absent, mild and moderate were assigned as mild. Similarly, discordant readings of absent, moderate and severe were assigned as moderate.

Airway wall thickening was quantified as the square-root wall area of a hypothetical airway with an internal perimeter of 10 mm (Pi10), which was calculated automatically from a large number of measures of airway wall thickness and lumen diameters throughout the lung [40]. Extent of emphysema was calculated as the percentage of lung volume with attenuation less than -950 Hounsfield units [HU] (LAA%-950) on inspiratory scans. Air trapping was calculated as the percentage of lung volume with attenuation less than -856 Hounsfield units [HU] (LAA%-856) on expiratory scans. Total lung volumes (TLV) and mean lung density (MLD) on both inspiratory (I) and expiratory (E) scans were measured, with additional parameters of air trapping calculated based on the ratio of expiratory/inspiratory measurements for both variables (E/I TLV ratio and E/I MLD ratio). Expiratory scans from 20 deployers could not be analyzed due to non-contiguous imaging, so air trapping parameters were unavailable for these images.

Quantitative HRCT was performed using Thirona LungQ software (Nijmegen, The Netherlands, http://www.thirona.eu). The airways were automatically extracted from inspiratory CT scans and visually approved by trained analysts. Airway wall thickness and lumen diameter quantification were extracted from cross-sections planes throughout the segmented airway tree without preselection of airway paths. Additional details on this analysis are presented in the online data supplement (Section A) of Charbonnier et al. [40].

All subjects were classified into an airway predominant, emphysema predominant, mixed (both airway and emphysema parameters abnormal), or normal phenotypes using quantitative imaging measurements as described below and in Table 3. Absent an established cut point in the published literature, we defined the airway predominant phenotype as Pi10 > median (2.26) among deployers, using an approach similar to that of Bodduluri et al. [15]. Predicted values for LAA%-950 and inspiratory TLV measured via HRCT quantitative analysis were calculated using MESA lung study equations [41]. Emphysema predominant phenotype was defined as ≥ 10% low attenuation areas based on LAA%-950. A cut point of 5% has been suggested for higher dose CT imaging that was typical with earlier generations of equipment, but recent work suggests that a greater cut point is more appropriate for reduced dose images, as were used in this study [15, 39].

### Statistical analysis

Demographic and clinical characteristics of all deployer and control study subjects were compared using Chi-square and Fisher Exact tests for categorical variables and t-tests for continuous variables using a Bonferroni correction to assess statistical significance. Additionally, comparisons between diagnosis groups (those with definite deployment lung disease, those with possible deployment-related distal lung disease, and controls) were made using Chi-square and Fisher Exact tests for categorical variables and ANOVA tests for continuous variables using a Bonferroni correction to assess statistical significance. Pairwise testing was performed for any variables where the overall test was statistically significant after Bonferroni correction.

We used linear regression models to compare quantitative imaging parameters between controls and deployers (overall and by diagnostic group) while adjusting for potential confounding variables. Previous literature has suggested that quantitative imaging measurements could differ by age, smoking status, smoking pack-years, sex, body mass index (BMI) and TLV [22, 40, 42, 43]. Control and deployer participants were successfully matched on smoking status so we did not consider this as a covariate. All other covariates were included in adjusted analyses a priori. Adjusted comparisons between groups were made using linear regression (PROC GLM) with dichotomous sex and continuous age, smoking pack-years (with a value of zero for non-smokers), and BMI. LAA%-950 and Pi10 measurements were also adjusted for HRCT measured inspiratory TLV. LAA%-856 was adjusted for measured expiratory TLV. Comparisons between diagnostic groups were Tukey adjusted to account for multiple comparisons.

We used linear regression to evaluate the relationship between pulmonary function parameters and quantitative imaging analysis measurements among deployers. We examined unadjusted results and results adjusted for smoking pack-years and BMI for consistency. LAA%-950 and Pi10 measurements were also adjusted for inspiratory TLV. LAA%-856 was adjusted for measured expiratory TLV. Continuous measures of FEV1_PP_ and the ratio of FEV1/forced vital capacity (FVC) were evaluated in all deployers. When available, RV_PP,_ RV/TLC ratio, and DLCO_PP_ were also analyzed in relation to quantitative imaging variables.

All analyses were performed in SAS v. 9.4.

## Results

A total of 82 deployer and 45 control chest HRCT images were included in analysis. Among deployers, 45 had definite deployment-related asthma and/or distal lung disease and 37 had possible disease. The median number of deployments reported was 2 (range 1–11) with a total mean duration across all deployments of 23.5 ± 20.7 months. As shown in Table 1, both study populations were predominantly male (85%) and were not statistically significantly different in terms of smoking status, with 66% overall having never smoked cigarettes, 27% reporting former smoking, and 7% current smokers. Deployers had 17.4 fewer mean smoking pack-years (p = 0.007) and were 16.1 years younger on average (p < 0.0001) than controls. Additionally, mean BMI for deployers was higher (p = 0.01) than that of controls (31.0 vs 28.1 kg/m^2^).

**Table 1:** Demographic and deployment characteristics and pulmonary function in controls and symptomatic deployers overall and by diagnostic sub-group

	Controls n = 45	Deployers n = 82	p-value*	Definite n = 45	Possible n = 37	p-value**
Demographic characteristics						
Age (years)	58.1 (± 6.7)	42.0 (± 10.2)	** < 0.0001**	39.4 (± 9.2)	45.2 (± 10.5)	** < 0.0001**^**abc**^
Male	37 (82%)	71 (87%)	0.51	40 (89%)	31 (84%)	0.65
Smoking status						
Never	28 (62%)	56 (68%)	0.74	33 (73%)	23 (62%)	0.77^+^
Former	13 (29%)	21 (26%)		10 (22%)	11 (30%)	
Current	4 (9%)	5 (6%)		2 (4%)	3 (8%)	
Pack-years	25.8 (± 21.8)	8.4 (± 13.0)	0.007	5.2 (± 3.6)	11.2 (± 17.3)	0.006
BMI (kg/m^2^)	28.1 (± 6.2)	31.0 (± 5.0)	0.01	30.4 (± 5.0)	31.6 (± 5.0)	0.01
Deployment characteristics						
Median (range) number of deployments	–	2 (1–11)	–	2 (1–11)	2 (1–7)	–
Total deployment duration (months)	–	23.5 (± 20.7)	–	20.1 (± 22.4)	24.7 (± 24.4)	–
Pulmonary function testing^						
FVC_PP_	94.7 (± 12.2)	91.7 (± 12.6)	0.20	91.0 (± 13.3)	92.7 (± 11.9)	0.36
FEV1_PP_	93.8 (± 14.8)	90.8 (± 15.1)	0.29	90.4 (± 17.1)	91.2 (± 12.3)	0.55
FEV1/FVC ratio (%)	76.0 (± 8.0)	78.8 (± 7.5)	0.05	79.4 (± 8.1)	78.1 (± 6.7)	0.11
RV_PP_	–	106.1 (± 20.9)	–	105.8 (± 20.4)	106.4 (± 21.9)	–
TLC_PP_	–	103.8 (± 12.4)	–	101.8 (± 11.2)	106.5 (± 13.5)	–
RV/TLC ratio (%)	–	30.9 (± 5.5)	–	30.8 (± 5.6)	31.0 (± 5.5)	–
DLCO_PP_	–	115.3 (± 20.6)	–	116.5 (± 20.3)	113.6 (± 21.1)	–

Bold values are statistically significant

Results are the number (%) or mean (± standard deviation) unless otherwise noted

*Deployers and controls were compared using t-tests for continuous variables (Satterthwaite result) and Chi-square tests for categorical variables. Statistically significant p-values are bolded if < 0.006, adjusting for multiple comparisons (8 tests) with a Bonferroni correction

**Diagnostic groups were compared using ANOVA for continuous variables and Chi-square or Fisher Exact tests (indicated by ^+^) for categorical variables. Statistically significant p-values are bolded if < 0.006, adjusting for multiple comparisons (8 tests) with a Bonferroni correction. Individual comparisons between groups were performed if the overall test was significant after Bonferroni correction with the following designations for significant differences (p < 0.05): ^a^definite vs controls, ^b^possible vs controls, ^c^definite vs possible

^One control is missing spirometry data. Spirometry values are all pre-bronchodilator and include the Forced Vital Capacity percent predicted (FVC_PP_), Forced Expiratory Volume in one second (FEV1_PP_), and the FEV1/FVC ratio. Residual volume percent predicted (RVpp) and total lung capacity percent predicted (TLC_PP_) were available for 76 deployers, and diffusion capacity for carbon monoxide percent predicted (DLCOpp) was available for 75 deployers

In unadjusted analyses (Table 1), spirometric measures (including FEV1_PP_, FVC_PP_ and FEV1/FVC) were not statistically significantly different between groups, with only 24 deployers (29%) and nine controls (21%) having any spirometric abnormality (FEV1, FVC, or FEV1/FVC < lower limit of normal). In the majority of deployers, RV_PP_ and DLCO_PP_ were normal, though 17 deployers had abnormally elevated lung volumes (RV_PP_ > 120_PP_) and two deployers had reduced diffusion capacity (DLCO_PP_ < 80_PP_). The mean duration between pulmonary function measurements and HRCT measurement for deployers was 5.5 ± 12.6 months. The mean duration between each participant’s last deployment and his or her HRCT was 7.5 ± 4.3 years.

On qualitative review (Table 2), only two deployers (3%) and two controls (5%) had findings of emphysema, while 23 deployers (28%) and seven controls (16%) had findings of bronchial wall thickening, and 27 deployers (33%) and 12 controls (27%) had findings of air trapping. None of these findings was significantly more common in either group. Unadjusted quantitative imaging analysis (Table 2) showed that the Pi10 parameter of airway wall thickening was significantly elevated (p < 0.0001) in deployers compared to controls. Deployers also had more emphysema (by LAA%-950) compared to controls, but this finding was not statistically significant (p = 0.06). All three parameters of air trapping (E/I TLV ratio, E/I MLD ratio, and expiratory LAA%-856) were significantly lower in deployers compared to controls, indicating that air trapping was not a common finding in symptomatic deployers.

**Table 2:** Qualitative consensus reads and quantitative imaging analysis measurements in controls and symptomatic deployers overall and by diagnostic sub-group

	Controls n = 45	Deployers^#^ n = 82	p-value*	Definite n = 45	Possible n = 37	p-value**
Radiologist consensus reads^						
Emphysema	2 (5%)	2 (3%)	0.61	2 (4%)	0	0.55
Bronchial wall thickening	7 (16%)	23 (28%)	0.13	13 (29%)	10 (27%)	0.28
Air trapping	12 (27%)	27 (33%)	0.55	21 (47%)	6 (16%)	0.01
General						
TLV_I_ (L)	6.1 (± 1.4)	6.2 (± 1.2)	0.85	6.1 (± 1.6)	6.2 (± 1.2)	0.86
TLV_E_ (L)	3.2 (± 0.7)	2.3 (± 0.5)	** < 0.0001**	2.2 (± 0.5)	2.4 (± 0.6)	** < 0.0001**^**ab**^
Emphysema						
LAA%-950 (%)	8.0 (± 6.3)	10.2 (± 5.9)	0.06	9.2 (± 5.6)	11.4 (± 6.2)	0.04
Airways disease/bronchial wall thickening						
Pi10 (mm)	1.9 (± 0.4)	2.3 (± 0.5)	** < 0.0001**	2.3 (± 0.6)	2.2 (± 0.4)	**0.0002**^**ab**^
Air trapping parameters						
E/I TLV Ratio	0.54 (± 0.12)	0.38 (± 0.07)	** < 0.0001**	0.37 (± 0.07)	0.38 (± 0.06)	** < 0.0001**^**ab**^
E/I MLD Ratio	0.84 (± 0.05)	0.74 (± 0.06)	** < 0.0001**	0.73 (± 0.06)	0.75 (± 0.07)	** < 0.0001**^**ab**^
LAA%-856 (%)	13.1 (± 10.5)	2.7 (± 4.1)	** < 0.0001**	1.9 (± 2.7)	3.9 (± 5.4)	** < 0.0001**^**ab**^

Bold values are statistically significant

Results are the count (percentage) or mean (± standard deviation)

^^^Radiologist consensus reads were not available for two controls and two deployers that were included in the analysis after original review by the radiology team

^#^Expiratory quantitative imaging values were not able to be calculated for 20 deployers

*Deployers and controls were compared using t-tests (Satterthwaite result) for continuous variables and Fisher’s exact tests for categorical variables. Statistically significant p-values are bolded if < 0.005, adjusting for multiple comparisons (10 tests) with a Bonferroni correction

**Diagnostic groups were compared using ANOVA for continuous variables and Fisher’s exact tests for categorical variables. Statistically significant p-values are bolded if < 0.005, adjusting for multiple comparisons (10 tests) with a Bonferroni correction. Individual comparisons between groups were performed if the overall test was significant after Bonferroni correction with the following designations for significant differences (p < 0.05): ^a^definite vs controls, ^b^possible vs controls, ^c^definite vs possible

**Fig. 2:**
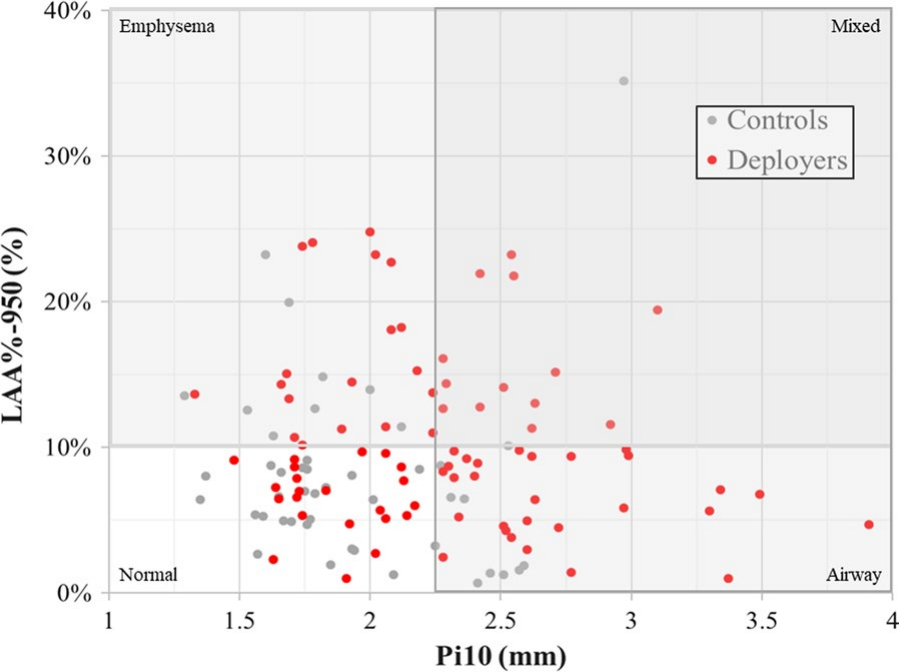
Deployers are more likely to cluster in the region with both airway disease (Pi10 > 2.26 mm) and/or emphysema (LAA%-950 ≥ 10%) than controls

As shown in Table 3, emphysema predominant (23%), airway predominant (34%), and mixed (16%) phenotypes were common among deployers, while all three phenotypes were significantly less common (p = 0.004) among controls (20%, 18%, and 4%, respectively). Figure 2 illustrates the distribution of Pi10 and LAA%-950 in deployers and controls.

**Table 3:** Interpretation of quantitative imaging parameters

Predominant phenotype	QI imaging parameter(s)	Interpretation	Criteria for abnormal	Controls n = 45	Deployers n = 82	p-value*
Emphysema	LAA%-950 (%)	Increases with extent of emphysema	≥ 10% low attenuation areas	9 (20%)	19 (23%)	**0.004**
Airway	Pi10 (mm)	Increases with extent of airway wall thickening	> median (2.26 mm)	8 (18%)	28 (34%)	
Mixed	LAA%-950 (%) Pi10 (mm)	As above for both parameters	≥ 10% low attenuation areas > median (2.26 mm)	2 (4%)	13 (16%)	
Normal	LAA%-950 (%) Pi10 (mm)	–	< 10% low attenuation areas ≤ median (2.26 mm)	26 (58%)	22 (27%)	
Air trapping	E/I TLV Ratio E/I MLD Ratio LAA%-856 (%)	Increase with extent of air trapping	No established cut points	–	–	–

Bold value is statistically significant

Results are the number (%)

*Deployers and controls were compared using Chi square tests

Adjusted mean differences in quantitative imaging parameters between deployers and controls are presented in Tables 4 and 5. While all of the quantitative measures examined were significantly different between deployers and controls in unadjusted analyses (Table 2), after adjustment for sex, age, smoking pack-years, BMI, and TLV (where appropriate), only LAA%-950, E/I TLV, and E/I MLD ratios remained statistically significant (p = 0.04, p < 0.0001, and p = 0.0008, respectively).

**Table 4:** Adjusted comparisons for quantitative imaging parameters of emphysema and airway wall thickening (n = 127)

	Adjusted^#^		
	Difference in means	95% confidence interval	p-value
LAA%-950 (%)			
Deployer vs control	2.95	0.18, 5.71	**0.04**
Definite vs control	1.80	− 1.34, 4.95	0.49^+^
Possible vs control	**3.67**	**0.75, 6.58**	**0.04**^+^
Definite vs possible	− 1.86	− 4.35, 0.62	0.30^+^
Pi10 (mm)			
Deployer vs control	0.19	− 0.03, 0.41	0.09
Definite vs control	0.23	− 0.03, 0.48	0.18^+^
Possible vs control	0.17	− 0.07, 0.40	0.34^+^
Definite vs possible	0.06	− 0.14, 0.26	0.82^+^

Bold values are statistically significant

^#^All measures were adjusted for sex, age, smoking pack-years, and body mass index. LAA%-950, Pi10, and LAA%-856 are also adjusted for total lung volume (inspiratory or expiratory as appropriate)

^+^Tukey p-value for multiple comparisons adjustment

**Table 5:** Adjusted comparisons of air trapping quantitative imaging parameters (n = 107)

	Adjusted^#^		
	Difference in means	95% Confidence interval	p-value
E/I TLV ratio			
Deployer vs control	**−** 0.11	**−** 0.16, **−** 0.06	** < 0.0001**
Definite vs control	**−** **0.11**	**−** **0.16, −** **0.05**	**0.0006**^+^
Possible vs control	**−** **0.11**	**−** **0.16, −** **0.06**	**0.0003**^+^
Definite vs possible	0.001	− 0.05, 0.05	1.00^+^
E/I MLD ratio			
Deployer vs control	− 0.05	− 0.08, − 0.02	**0.0008**
Definite vs control	**−** **0.05**	**−** **0.09, −** **0.02**	**0.004**^+^
Possible vs control	**−** **0.05**	**−** **0.08, −** **0.01**	**0.01**^+^
Definite vs possible	− 0.008	− 0.04, 0.02	0.83^+^
LAA%-856 (%)			
Deployer vs control	− 1.1	− 4.4, 2.2	0.51
Definite vs control	− 1.0	− 4.7, 2.7	0.86^+^
Possible vs control	− 1.2	− 4.7, 2.4	0.79^+^
Definite vs possible	0.17	− 2.9, 3.3	0.99^+^

Bold values are statistically significant

^#^All measures were adjusted for sex, age, smoking pack-years, and body mass index. LAA%-950, Pi10, and LAA%-856 are also adjusted for total lung volume (inspiratory or expiratory as appropriate)

^+^Tukey p-value for multiple comparisons adjustment

Comparing controls to deployers who did not meet clinical criteria for definite lung disease, those with possible disease were significantly (p = 0.04) more likely to have emphysema based on LAA%-950, even after adjustment. In unadjusted analyses, these cases of possible deployment-related distal lung disease were also significantly more likely to have airway wall thickening, though adjustment diminished this association. These findings are notable, since asthma had been excluded for all with possible disease, but none had undergone lung biopsy to assess distal lung histologic abnormalities. As in deployers with definite lung disease (asthma, biopsy-proven distal lung disease or both), those with possible disease were less likely to have findings of air trapping than controls.

We also examined which demographic and lung volume characteristics were associated with each of the quantitative imaging measures. Parameter estimates for fully adjusted linear regression models are included in Tables 6 and 7. LAA%-950 was significantly positively associated with TLV (p < 0.0001). Pi10 was significantly negatively associated with age, but positively associated with smoking pack-years and BMI. TLV was modestly (but not significantly) negatively associated with Pi10. The E/I TLV and E/I MLD ratios were significantly positively associated with age and negatively associated with BMI. While sex, age, smoking pack-years, and BMI were modestly associated with LAA%-856, the most strongly associated covariate was expiratory TLV, which was significantly positively associated with LAA%-856 and explains most of the observed difference in air trapping between deployers and controls.

**Table 6:** Parameter estimates from multiple linear regression analyses for emphysema and airway quantitative imaging measurements (n = 127)

Variable	Comparison group or increase	Parameter estimate [95% confidence interval] (p-value)*	
		Change in LAA%-950 (%)	Change in Pi10 (mm)
Intercept	**–**	− 4.7 [− 14.0, 4.5]	2.5 [1.7, 3.2]
Deployer	Control	**2.9 [0.2, 5.7]** **(p = 0.04)**	0.2 [− 0.03, 0.4] (p = 0.09)
Male	Female	− 0.4 [− 3.5, 2.6] (p = 0.78)	0.03 [− 0.2, 0.3] (p = 0.82)
Age	+ 1 year	0.02 [− 0.09, 0.1] (p = 0.72)	− **0.01 [**− **0.02, **− **0.002] (p = 0.02)**
Smoking pack-years	+ 1 pack-year	0.04 [− 0.04, 0.1] (p = 0.35)	**0.008 [0.002, 0.01] (p = 0.01)**
BMI	+ 1 kg/m^2^	− 0.07 [− 0.3, 0.1] (p = 0.44)	**0.02 [0.0003, 0.03] (p = 0.045)**
TLV (inspiratory)	+ 1 L	**2.2 [1.3, 3.1]** **(p < 0.0001)**	− 0.07 [− 0.1, 0.004] (p = 0.07)

Bold values are statistically significant

*Results are the p-value from the F-test using a linear model with all covariates included at the same time

**Table 7:** Parameter estimates from multiple linear regression analyses for air trapping quantitative imaging measurements (n = 107)

Variable	Comparison group or increase	Parameter estimate [95% confidence interval] (p-value)*		
		Change in E/I TLV ratio	Change in E/I MLD ratio	Change in LAA%-856 (%)
Intercept		0.4 [0.3, 0.6]	0.7 [0.7, 0.8]	**−** 10.6 [**−** 20.6, **−** 0.6]
Deployer	Control	**−** **0.01 [−** **0.2, −** **0.06]** **(p < 0.0001)**	**−** **0.05 [−** **0.08, −** **0.02] (p = 0.0008)**	**−** 1.1 [**−** 4.4, 2.2] (p = 0.51)
Male	Female	**−** 0.004 [**−** 0.05, 0.04] (p = 0.87)	**−** 0.002 [**−** 0.03, 0.02] (p = 0.90)	**−** 3.0 [**−** 6.2, 0.3] (p = 0.07)
Age	+ 1 year	**0.003 [0.001, 0.005]** **(p = 0.003)**	**0.003 [0.002, 0.004]** **(p < 0.0001)**	0.1 [**−** 0.01, 0.3] (p = 0.07)
Smoking pack**−** years	+ 1 pack**−** year	**−** 0.0006 [**−** 0.002, 0.0008] (p = 0.39)	**−** 0.0001 [**−** 0.0009, 0.0006] (p = 0.73)	0.07 [**−** 0.01, 0.2] (p = 0.09)
BMI	+ 1 kg/m^2^	**−** 0.002 [**−** 0.006, 0.0007] (p = 0.13)	**−** **0.003 [−** **0.005, −** **0.0008]** **(p = 0.006)**	**−** 0.2 [**−** 0.4, 0.05] (p = 0.13)
TLV (expiratory)	+ 1 L	–	–	**6.9 [4.7, 9.0] (p < 0.0001)**

Bold values are statistically significant

*Results are the p-value from the F-test using a linear model with all covariates included at the same time

As expected, markers of abnormal airway findings on quantitative imaging were associated with more abnormal lung function parameters. Markers of obstruction, both FEV1_PP_ and FEV1/FVC ratio significantly declined with increasing Pi10, and FEV1/FVC ratio significantly decreased with increasing LAA%-950 (Table 8). FEV1_PP_ and FEV1/FVC were also significantly negatively associated with the E/I TLV and E/I MLD ratios (Table 9). Additionally, more abnormal (higher) RV_PP_ was associated with increased Pi10 (Table 8). All three imaging measures of air trapping (E/I TLV ratio, E/I MLD ratio, and LAA%-856) were positively associated with lung function parameters of air trapping including higher RV_PP_ and RV/TLC ratio (Table 9). We found no observable association between quantitative HCRT measurements and DLCO_PP_.

**Table 8:** Relationship between emphysema and airway quantitative imaging analysis parameters and pulmonary function measurements among deployers

Variable	n	Estimated change βa [95% CI]	
		+ 1% LAA%-950	+ 1 mm Pi10
FEV1_PP_	82	**−** 0.4 [**−** 1.0, 0.2]	**−** **14.1 [−** **19.6, −** **8.5]**
FEV1/FVC	82	**−** **0.4 [−** **0.7, −** **0.05]**	**−** **8.6 [−** **11.5, −** **5.7]**
RV_PP_	76	0.4 [**−** 0.5, 1.3]	**11.6 [2.4, 20.9]**
RV/TLC	76	0.2 [**−** 0.04, 0.4]	1.7 [**−** 0.7, 4.2]
DLCO_PP_	75	**−** 0.8 [**−** 1.8, 0.06]	0.6 [-9.4, 10.7]

Bold values are statistically significant

a = All measures were adjusted for smoking pack-years and body mass index. LAA%-950, Pi10, and LAA%-856 are also adjusted for total lung volume (inspiratory or expiratory as appropriate)

**Table 9:** Relationship between air trapping quantitative imaging analysis parameters and pulmonary function measurements among deployers

Variable	n	Estimated change βa [95% CI]		
		+ 0.1 E/I TLV Ratio	+ 0.1 E/I MLD Ratio	+ 1% LAA%-856
FEV1_PP_	62	**−** **9.9 [−** **15.6, −** **4.1]**	**−** **7.2 [−** **13.5, −** **0.9]**	**−** 0.8 [**−** 2.1, 0.5]
FEV1/FVC	62	**−** **4.9 [−** **7.7, −** **2.2]**	**−** **4.9 [−** **7.8, −** **2.0]**	**−** 0.3 [**−** 0.9, 0.3]
RV_PP_	59	**9.5 [0.8, 18.3]**	**12.3 [3.4, 21.2]**	**−** 0.1 [**−** 1.8, 1.6]
RV/TLC	59	**5.0 [3.0, 6.9]**	**4.6 [2.5, 6.7]**	0.4 [**−** 0.06, 0.9]
DLCO_PP_	58	2.4 [**−** 7.0, 11.8]	3.9 [**−** 5.8, 13.7]	**−** 1.3 [**−** 3.1, 0.6]

Bold values are statistically significant

a = All measures were adjusted for smoking pack-years and body mass index. LAA%-950, Pi10, and LAA%-856 are also adjusted for total lung volume (inspiratory or expiratory as appropriate)

## Discussion

In symptomatic military deployers with clinically confirmed asthma and/or biopsy-proven distal lung disease, low-dose chest HRCT quantitative emphysema measurement is useful in detecting subtle abnormalities typically not found on visual imaging assessment or pulmonary function testing. Deployers had significantly more emphysema (by LAA%-950) than controls, even after adjusting for multiple potentially confounding variables. Importantly, we found that symptomatic deployers in whom a diagnosis of asthma was excluded and who did not undergo lung biopsy had abnormal quantitative imaging parameters of emphysema (p = 0.04). Lung biopsy is an invasive procedure with attendant risks. Quantitative imaging may have particular utility as a noninvasive marker of distal lung disease, with important implications for both diagnosis and management in this patient population with persistent and often disabling respiratory symptoms.

Bronchial wall thickening (by Pi10) was higher in symptomatic deployers compared to controls in unadjusted analyses and remained elevated, though nonsignificant, in adjusted analyses. This failure to detect a difference between deployers and controls may be a limitation of the control group available.

In contrast to findings of emphysema and airway wall thickening, quantitative markers of abnormal air trapping were more common on control images than deployer images. This is likely explained by differences in both age and cumulative smoking between groups. Several studies have shown that the frequency and extent of air trapping increase with age [44, 45], and deployers were significantly younger than controls. Moreover, while the majority of both deployers and controls in this study were never smokers, deployers who had smoked had substantially fewer total pack-years. Previous investigators have shown a significant increase in air trapping extent on quantitative imaging with increasing smoking history [46].

Quantitative imaging findings of airways disease were also associated with lung physiologic parameters of obstruction. While spirometry is often normal or non-diagnostic in symptomatic deployers, the presence of bronchial wall thickening by Pi10 was significantly inversely related to FEV1_PP_ and FEV1/FVC in the deployer group overall. This inverse relationship is consistent with findings from patients with COPD [24, 25, 40] and similar in magnitude to the entire COPDGene diseased population analyzed [40]. Further, among symptomatic deployed military personnel, the FEV1/FVC ratio was strongly correlated with imaging parameters of emphysema, indicating the potential utility of LAA%-950 in detecting early emphysema even in a population with largely normal spirometry. We also found that bronchial wall thickening (by Pi10) was associated with higher RV_PP_, a marker of hyperinflation and air trapping. While not statistically significant in our study, we found that LAA%-950 was negatively associated with DLCO_PP_ as has been observed in patients with COPD [22].

A number of investigators have shown that both abnormal Pi10 and imaging markers of emphysema are associated with subsequent development of airflow limitation in subjects without spirometric limitation at baseline, indicating that clinical follow-up of this deployed population is important [47]. In the MESA cohort, Pi10 was associated with accelerated lung function decline and increased risk of incident COPD and chronic lower respiratory disease (CLRD) hospitalizations and mortality, independent of initial lung function among participants without clinical lung disease at baseline [48]. Our findings may be helpful in predicting prognosis and guiding clinical management of military personnel with respiratory symptoms following deployment. Additionally, quantitative HRCT findings may inform or enhance the diagnostic value of newer non-invasive markers of deployment-related lung disease such as the lung clearance index score from multiple breath washout testing [49] or measures of resistance and reactance using impulse oscillometry [43].

Our study has several limitations. First, demographic differences between deployers and controls (with controls being significantly older and having more smoking pack-years, and deployers having higher BMIs) may have limited our ability to detect imaging differences between groups. Second, imaging techniques can vary between sites, and technicians and quantitative measures can even vary within a subject based on the size of the breath the subject is able to take during a given scan. This could have resulted in some measurement error, though this possibility was likely reduced by the use of the same image acquisition protocols and consistent training of staff between studies and sites. Third, with no established cut-point for Pi10 in the published literature, classifying airways predominant disease using our own study population may limit the reliability of this threshold-based phenotype in other populations. Fourth, quantitative imaging analysis using these techniques is not widely available and requires substantial technical knowledge and expertise. Despite these limitations, our study is the first to demonstrate the potential utility of quantitative analysis of HRCT in a population of symptomatic military deployers with large and small airways disease, with important implications for diagnosis and management.

## Conclusion

In summary, analysis of quantitative imaging parameters of emphysema and airway wall thickening identifies subtle abnormalities that may be useful in noninvasive diagnosis of deployment-related lung disease in a population where lung function is typically normal.

## Abbreviations

ALD: Adjusted lung density
ATS: American Thoracic Society
BMI: Body mass index
BODE: Body-mass, airflow Obstruction, Dyspnea, and Exercise
CLRD: Chronic lower respiratory disease
COPD: Chronic obstructive pulmonary disease
CPET: Cardiopulmonary exercise testing
E: Expiratory
DLCO_PP_: Diffusion capacity for carbon monoxide percent predicted
DLD: Deployment-related lung disease
DDLD: Deployment-related distal lung disease
DRA: Deployment-related asthma
FVC_PP_: Forced Vital Capacity percent predicted
FEV1_PP_: Forced Expiratory Volume in one second
GLI: Global Lung Function Initiative
HRCT: High resolution computed tomography
I: Inspiratory
LAA%-950: The percentage of low-attenuation lung area below -950 Hounsfield units on inspiratory scan
LAA%-856: The percentage of low-attenuation lung area below -856 Hounsfield units on expiratory scan
LCI: Lung clearance index
Pi10: Airway wall thickness expressed as the square root of wall area at the airways with a perimeter of 10 mm
RV_PP_: Residual volume percent predicted
SGRQ: St. George's Respiratory Questionnaire
TLV: Total lung volume
VDGF: Vapors, dusts, gases, and fumes
VO2max_PP_: Maximal oxygen consumption

## Declarations

### Availability of data and materials

The datasets used and/or analyzed during the current study are available from the corresponding author on reasonable request.
